# Branding Practices on Four Dairies in Kantale, Sri Lanka

**DOI:** 10.3390/ani8080137

**Published:** 2018-08-07

**Authors:** Sarah J. J. Adcock, Cassandra B. Tucker, Gayani Weerasinghe, Eranda Rajapaksha

**Affiliations:** 1 Center for Animal Welfare, Department of Animal Science, University of California, Davis, CA 95616, USA; sadcock@ucdavis.edu; 2 Department of Veterinary Clinical Sciences, Faculty of Veterinary Medicine and Animal Science, University of Peradeniya, Peradeniya 20400, Sri Lanka; gayaniweerasinghe86@gmail.com (G.W.); earajapaksha@gmail.com (E.R.)

**Keywords:** animal welfare, cattle, hot-iron branding, ear tags, smallholders

## Abstract

**Simple Summary:**

Branding cattle with hot irons is a painful procedure, inflicting severe burns that take weeks to heal. Although Sri Lanka prohibits hot-iron branding, the practice is still common in some areas of the country but has not been described. We observed branding practices on four smallholder farms and identified welfare concerns and challenges impeding adoption of alternative methods of identification, such as ear tags. Farmers used multiple irons to mark their initials and, in some cases, their address, with the largest brands extending across the ribs and hip. Farmers did not consider ear tags a viable alternative to hot-iron branding because of issues with security and tag retention. Hot-iron branding raises serious animal welfare concerns and efforts to introduce more welfare-friendly alternatives are needed.

**Abstract:**

Hot-iron branding is illegal in Sri Lanka, but is still commonly used to identify dairy herds in extensive farming systems, which are primarily located in the country’s Dry Zone. Despite the negative welfare implications of this practice, there is no written documentation of branding in this region. We observed branding on four smallholder farms in Kantale, Eastern Province to understand the welfare implications associated with the procedure and challenges limiting the uptake of more welfare-friendly alternatives, such as ear tagging. Areas of welfare concern included the duration of restraint, the size and location of the brand, and the absence of pain relief. Animals were restrained with rope for an average duration of 12 min (range 8–17 min). Farmers used multiple running irons to mark their initials and, in some cases, their address, with the largest brands extending across the ribs and hip. Three farmers applied coconut or neem oil topically to the brand after performing the procedure. No analgesics were given before or after branding. Farmers reported that poor ear tag retention in extensive systems and theft were the main factors impeding the uptake of alternative forms of identification. Branding is also practiced as part of traditional medicine in some cases. Given the clear evidence that hot-iron branding impairs animal welfare and there is no evidence that this can be improved, alternative identification methods are needed, both in Sri Lanka, as well as in other countries engaging in this practice.

## 1. Introduction

In many countries, including Sri Lanka, cattle herds have traditionally been identified by hot-iron branding, a procedure that permanently marks the animal via thermal damage to the skin. Hot-iron branding produces acute pain [1,2,3,4,5]. The resulting burns take at least 10 weeks to heal and are more sensitive than unbranded tissue throughout that time [6,7]. In response to increasing animal welfare concerns over this practice, several countries have prohibited its use in cattle [8,9], and horses [10]. In 2017, the Sri Lankan government amended the Animals Act, No. 29 of 1958, to declare hot-iron branding illegal.

Sri Lanka has successfully transitioned to ear tagging in intensive and semi-intensive dairy practices, the majority of which are owned by smallholder farmers. However, hot-iron branding is still prevalent in the Eastern Province, which accounts for approximately 20% of the country’s one million cattle [11]. Cattle are primarily managed extensively in this region, grazing on communal pasture lands. Herds average approximately 25 cattle [12], and typically comprise a mix of zebu and Jersey crosses.

No written records exist on branding practices in the Eastern Province. To describe this practice and identify potential welfare concerns, we observed four live branding events in Kantale, a town in the Trincomalee District of the Eastern Province, in May 2017. We discuss the welfare implications of this procedure and the challenges impeding adoption of ear tags.

## 2. Description of Branding Practices

We observed four smallholder dairy farmers while they each branded one of their animals. Farmers were selected using convenience sampling based on their willingness to participate in the study. Cattle were kept on communal grazing lands with minimal inputs, as is typical for the region. Four observers attended each branding (one branding/farm = 4 brandings total). The procedure was observed from the time the animal was caught until she was released. Table 1 summarizes the branding practices observed on the farms. Photos were taken with the farmer’s consent. Since branding is prohibited in Sri Lanka, the farmers requested that no photos be taken during the procedure itself.

## 3. Welfare Implications

### 3.1. Restraint and Branding Methodology

**Figure 1:**
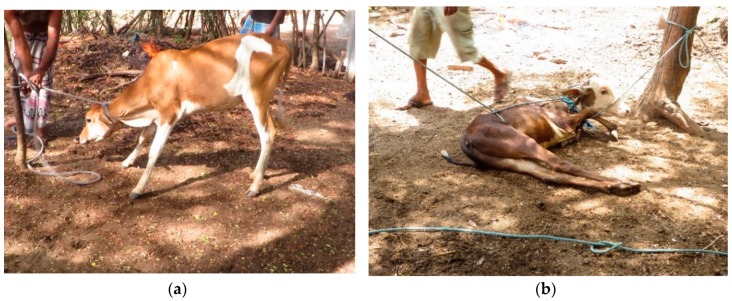
In preparation for branding, animals were roped around the neck (**a**) and laid on the ground (**b**).

**Figure 2:**
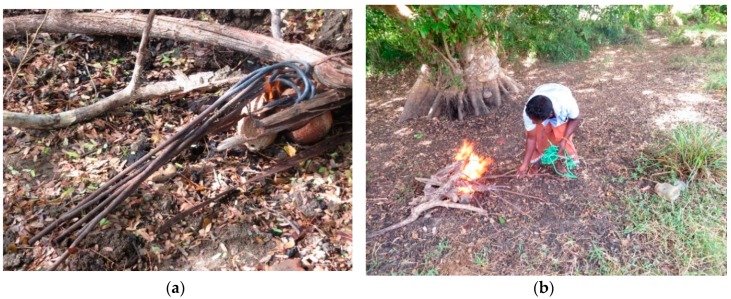
Fire-heated irons used for branding on Farm 1 (**a**) and Farm 4 (**b**).

The method of restraint was similar across farms; after the animal was separated from the herd, she was roped to a tree and laid on the ground (Figure 1). Brands consisted of a series of sequential burns from multiple running irons heated over an open fire (Figure 2). An iron was applied multiple times to the same area until a satisfactory burn was achieved or the iron lost its heat. More applications were needed for irons that cooled faster, prolonging the period of restraint. Similarly, larger brands took longer since they were made up of more individual marks. All animals vocalized when the iron was first applied. Other evidence suggests that cattle find handling and restraint aversive [13], and minimizing branding time could reduce stress arising from the procedure. In countries where branding is legal, like the U.S. and Australia, pre-shaped irons are used to stamp the brand rather than draw it. From a welfare perspective, stamping is preferable over drawing, as less time is needed to apply the brand. Although precise application times were not measured in the current study, all brands took at least 1 min to apply, substantially longer than the 1–5 s reported for stamp brands in horses and cattle [7,14,15].

### 3.2. Brand Size and Location

**Figure 3:**
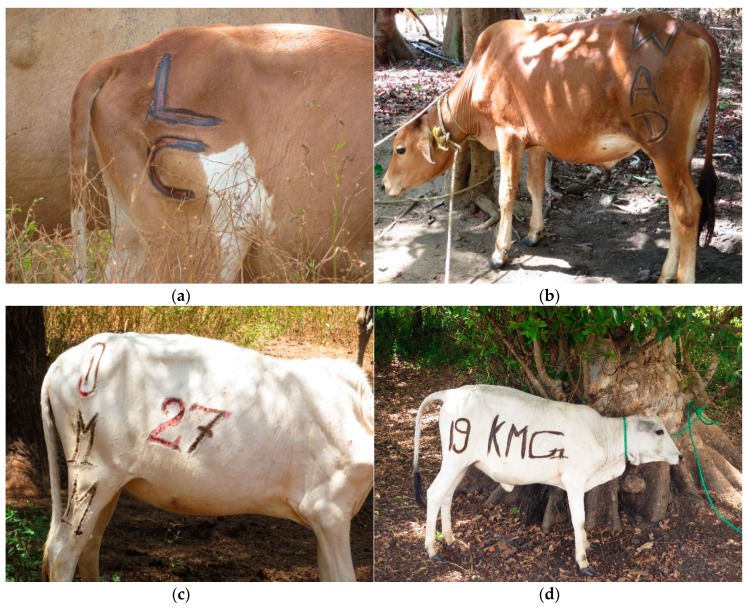
Brand size and location on Farm 1 (**a**), Farm 2 (**b**), Farm 3 (**c**), and Farm 4 (**d**). For Farm 3, the calf fled immediately after branding and we were unable to photograph her, so (**c**) shows an animal branded one week earlier from the same farm.

Brand size and location varied substantially (Figure 3). Brands containing both the owner’s initials and address took up the most space, extending across the ribs and the hip (Farms 3 and 4). Although no study has investigated the relative pain experienced from branding different body regions, there is evidence in humans that tissue damage from thermal injury increases with decreasing skin thickness [16]. Thus, it is probable that brands over the rib and upper hindleg are more painful than brands on the hip, since the skin is thinner in the former locations [17]. Additionally, sensitivity may decrease with age, as skin continues to thicken for several years following birth [18]. However, further research is needed to fully understand the welfare consequences of performing painful procedures at different ages [19].

The location of the brand may also influence healing time. Brands on the hip take at least 10 weeks to heal [6,7]. In goats, excision wounds on the dorsal trunk of the animal healed faster than those on the upper hindleg [20]. The authors suggest that the slower healing observed in leg wounds was due to the relatively poorer vascularization, increased contact with ground, joint movement, and minimal soft tissue between skin and bone compared to the dorsal trunk. Thus, it is conceivable that healing times may be longer for brands on the ribs and leg than those on the hip, although this has not been tested. Brands located over joints may also result in a persistent inability to perform full range of motion due to wound and scar contracture, which is a common complication seen in human burn patients [21]. In the U.S., the vast majority of brands are located on the hip, primarily to reduce economic losses from hide damage to high-value areas like the rib [22].

The brands we observed were substantially larger than U.S. brands. In 2005, hot-iron cattle brands in the U.S. averaged 24.7 × 24.7 cm for rib brands, 14.9 × 14.9 cm for hip brands, and 17.1 × 17.1 cm for shoulder brands [22]. Increasing total burned surface area is a major risk factor for chronic pain, impaired physical function, sleep problems, and mortality in humans [23,24,25]. In addition to these potential increased risks, larger brands may also increase the probability of squamous cell carcinoma, which has been reported to develop on brand sites in cattle and sheep [26,27].

### 3.3. Pain Relief

**Figure 4:**
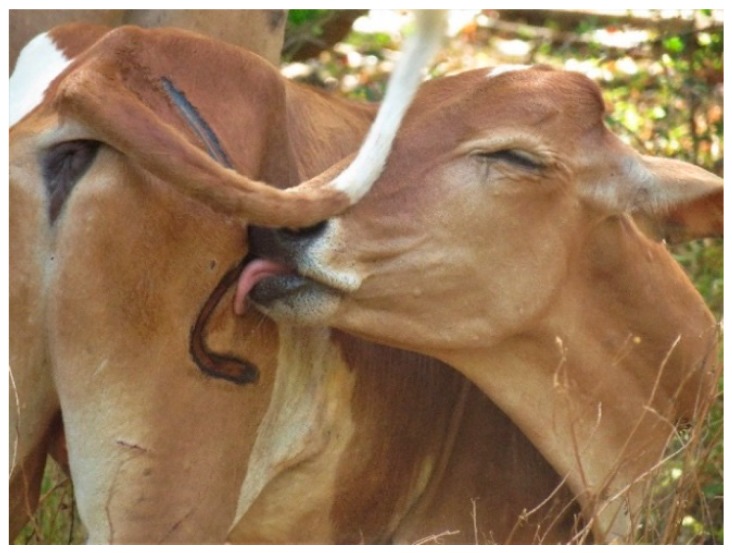
Animals were observed licking the brand immediately after the procedure.

Branding was performed without any anesthesia on all farms, as is standard practice worldwide. In Sri Lanka, branding is prohibited and therefore is done without veterinary intervention. However, in countries where branding is permitted and, in some cases, required by law, lack of appropriate analgesics to manage branding pain, the financial and time constraints involved with administering analgesia, and restricted access to drugs impede the uptake of pain relief. Branding unequivocally produces acute pain, but could also conceivably produce long-lasting states of pain spanning several months or years, as has been seen in human patients suffering from third-degree burn injuries, as reviewed previously [19]. There are no established methods for treating long-term pain in cattle. In the current study, three of the farmers applied coconut or neem oil topically to the wound. Two of these farmers added turmeric for its perceived healing and anti-bacterial benefits. Coconut oil, neem oil, and curcumin, the active compound in turmeric, have been shown to hasten wound healing in rats [28,29,30]; however, in these studies the treatment was applied at multiple timepoints, and it is unclear whether a one-time application would have any benefit. Moreover, we observed wound-licking immediately after the procedure, which could limit the efficacy of topical preparations (Figure 4). Neither a cooling gel, applied once or twice after branding, nor a single injection of flunixin meglumine, improved healing or pain outcomes [6,7]. Due to a lack of feasible, cost-effective, and long-lasting treatments, the only viable approach to pain management currently is a preventive one, in which more welfare-friendly alternatives to branding are employed.

## 4. Constraints to Uptake of Ear Tagging

All farmers were aware that branding is both painful and illegal and wanted to adopt a more welfare-friendly alternative. Ear tags were introduced on the farms by the herd veterinarian; however, the farmers identified some factors impeding their widespread use.

### 4.1. Extensive Management System

**Figure 5:**
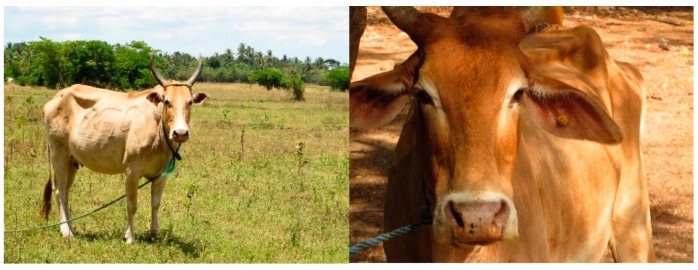
Farmers reported that efforts to introduce ear tags in the Eastern Province are impeded by poor tag retention in extensive systems.

The farmers believed ear tags were poorly designed for extensive operations; in addition to hindering identification from a distance, the farmers reported that ear tags were easily dislodged (Figure 5).

### 4.2. Theft

Brands are reported to act as a theft deterrent, while ear tags are perceived as easily removed. However, branding does not provide absolute proof of ownership. One farmer recounted how law enforcement was unable to intervene after thieves stole and rebranded her high-producing cow. Although the Agricultural Insurance Board offers livestock insurance against theft, coverage is limited due to poor extension services [31].

### 4.3. Traditional Medicine

**Figure 6:**
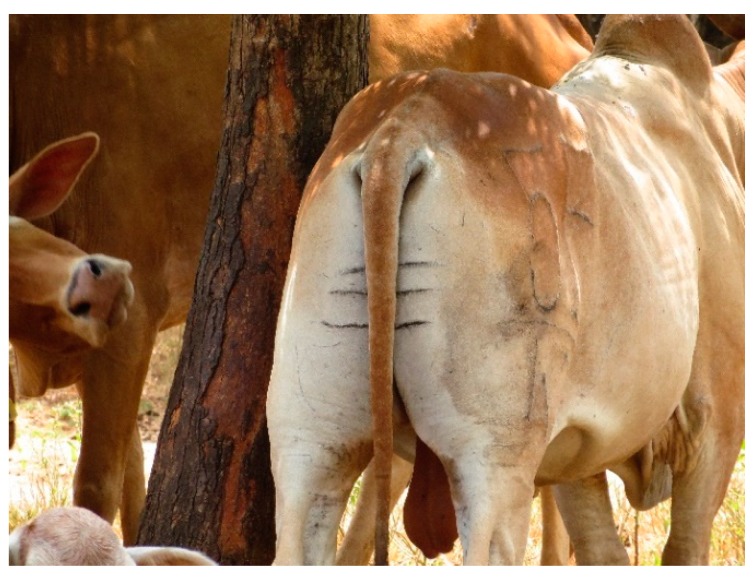
Branding is practiced in traditional veterinary medicine. Here, the three lines branded along either side of the tail are meant to guard against disease.

In the past, branding was performed to prevent and treat various diseases in Sri Lankan dairy herds [32]. Nowadays, farmers practice branding strictly for identification purposes, but one farmer explained that he brands for medicinal reasons as well. We observed one cow, belonging to one of the Sri Lanka Army herds, with brand marks intended to stave off disease (Figure 6).

## 5. Alternatives

Due mainly to issues regarding security and tag retention, ear tags were not considered a viable alternative to hot-iron branding by the farmers we observed in Sri Lanka’s Eastern Province. Tag retention is likely determined by several factors such as tag position, size, application, infection rate, tag design, environmental conditions, length of time since application, and on-farm management [33]. In Canada, the radio frequency identification (RFID) tag retention rate for mature cows averaged 18% loss over 4 years [34]. Ear tags perform variably in small ruminants, with losses ranging from 0 to 20% [35,36,37]. In addition, the acute stress response to ear tagging and possible long-term ear damage raise welfare concerns, albeit minor compared to those associated with hot-iron branding [38,39].

Injectable transponders and electronic rumen boluses may present a more secure, long-lasting identification method. With some exceptions, these devices allow reliable identification (>98%) in cattle [40], horses [41], camels [42], and goats [35]. In contrast, the readability of hot-iron brands in cattle is unknown. Few studies have evaluated the welfare implications of boluses and injectable transponders. In horses, microchip transponder injection causes less pain and inflammation than hot-iron branding [43]. However, another study reported mild to severe inflammation at transponder implantation sites in 5 of 16 horses [44]. Recently, biometric identification (e.g., nose prints, DNA profiling, iris scanning, and retinal scanning) has been investigated as a non-invasive tool that is less prone to fraud than the aforementioned alternatives [45].

There is little information on farmers’ opinions about identification technologies, but a survey of UK sheep farmers indicates that beliefs about the practicality and usefulness of electronic technology play an important role in its uptake [46]. Thus, communicating the management benefits of electronic identification tools will enhance farmers’ trust in the technology and its subsequent adoption. For boluses and injectable transponders, the lack of visibility, although improving security, may be perceived adversely by farmers [47]. To overcome this potential barrier, a combination of RFID and biometric technologies may be ideal.

## 6. Conclusions

Given the current research on welfare implications of branding and the well-documented long-term ramifications of severe burns in humans, we do not believe that this procedure can be performed humanely under any circumstances. We recommend that research efforts focus on developing, adapting, and applying modern identification technologies that are long-lasting, cost-effective, and welfare-friendly. Surveying farmers on their attitudes towards animal welfare and branding alternatives will inform the approach needed to successfully introduce humane identification methods. Concurrently, increased government support for veterinary and extension services will help to raise farmer awareness of the welfare concerns associated with branding while alternatives are explored.

## Figures and Tables

**Table 1:** Record of hot-iron branding practices on four smallholder farms in Kantale, Eastern Province, Sri Lanka.

Measure	Farm			
	1	2	3	4
**Age of animal (months)**	8	18	7	18
**Restraint method**	Rope—legs not tied	Rope—legs not tied	Rope—legs tied	Rope—legs tied
**Restraint duration (min)**	8	11	13	17
**Symbol**	LC	WAD	DMM 27	19 KMG
**Location of brand**	Right upper and lower hip	Left upper and lower hip	Right upper and lower hip, rib, upper hindleg	Right upper and lower hip, rib
**Pre-branding treatment**	None	None	None	None
**Post-branding treatment**	Coconut oil & turmeric	Coconut oil, neem oil & turmeric	Coconut oil	None
